# Two New Quinochalcone *C*-Glycosides from the Florets of *Carthamus tinctorius*

**DOI:** 10.3390/ijms150916760

**Published:** 2014-09-22

**Authors:** Shijun Yue, Yuping Tang, Chengmei Xu, Shujiao Li, Yue Zhu, Jin-Ao Duan

**Affiliations:** 1 Jiangsu Collaborative Innovation Center of Chinese Medicinal Resources Industrialization, Nanjing University of Chinese Medicine, Nanjing 210023, China; E-Mails: shijun_yue@163.com (S.Y.); xurealise@sina.com (C.X.); 18795965099@163.com (S.L.); nzyzy808@163.com (Y.Z.); dja@njutcm.edu.cn (J.-A.D.); 2 Jiangsu Key Laboratory for High Technology Research of TCM Formulae, Nanjing University of Chinese Medicine, Nanjing 210023, China; 3 National and Local Collaborative Engineering Center of Chinese Medicinal Resources Industrialization and Formulae Innovative Medicine, Nanjing University of Chinese Medicine, Nanjing 210023, China

**Keywords:** *Carthamus tinctorius*, *Hong hua*, quinochalcone *C*-glycosides, hydroxysafflor yellow B, hydroxysafflor yellow C, anti-oxidative activity

## Abstract

Two new quinochalcone *C*-glycosides, named hydroxysafflor yellow B (**1**) and hydroxysafflor yellow C (**2**), along with two known quinochalcone *C*-glycosides, safflomin C (**3**) and saffloquinoside C (**4**), and one known flavanone, (2*R*)-4',5-dihydroxyl-6,7-di-*O*-β-d-glucopyranosyl flavanone (**5**), were isolated from the florets of *Carthamus tinctorius*. Their structures were determined by extensive spectroscopic (UV, IR, HR-ESI-MS, 1D and 2D NMR) analyses. In addition, these quinochalcone *C*-glycosides together with hydroxysafflor yellow A and anhydrosafflor yellow B were evaluated for their anti-oxidative effects against H_2_O_2_-induced cytotoxicity in cultured H9c2 cells. Among them, compound **2** exhibited significant anti-oxidative effects.

## 1. Introduction

**Figure 1:**
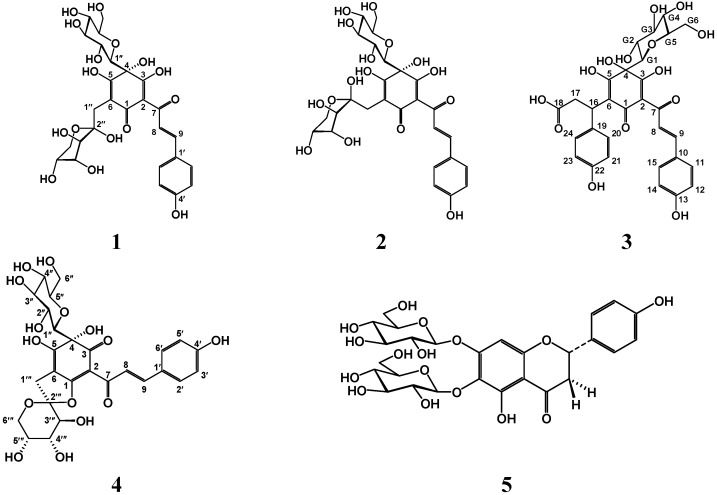
The chemical structures of compounds **1**–**5** isolated from *Carthamus tinctorius*.

The florets of *Carthamus tinctorius*, which is a common traditional Chinese medicine (TCM), are known as *Hong hua*, safflower. In traditional Chinese medicinal prescription, safflower has mainly been taken as decoction to treat stroke, coronary heart disease and angina pectoris for thousands of years [1]. Phytochemical and pharmacological research has shown that the water soluble components are responsible for the therapeutic effects, especially quinochalcone *C*-glycosides, which are regarded as the main active and characteristic compounds. Prior to this study, 18 quinochalcone *C*-glycosides have been obtained from safflower. Among them, hydroxysafflor yellow A (HSYA), the main active component of safflor yellow, has been demonstrated to restrain the conglomeration of platelets, promote blood circulation, remove blood stasis, anti-oxidation, and promote metabolism [2]. Through an antioxidant-oriented approach, our group have isolated and identified five major components from water extract of safflower as 6-hydroxykaempferol 3,6,7-tri-*O*-β-d-glucoside, 6-hydroxykaempferol 3-*O*-β-rutinoside-6-*O*-β-d-glucoside, 6-hydroxykaempferol 3-*O*-β-d-glucoside, HSYA and anhydrosafflor yellow B [3]. On this basis we then successfully isolated two new quinochalcone *C*-glycosides, named hydroxysafflor yellow B (**1**) and hydroxysafflor yellow C (**2**), together with the three known ones. Their structures are depicted in Figure 1. Herein, we mainly describe the isolation and structural elucidation of the new compounds.

## 2. Results and Discussion

### 2.1. Results

Compound **1** was obtained as a yellow powder. The IR spectrum of **1** showed the presence of hydroxyl groups (3445 cm^−1^), carbonyl groups (1636 cm^−1^), and aromatic rings (1512 cm^−1^). A molecular formula of C_27_H_32_O_16_ was deduced on the basis of high resolution electrospray ionization mass spectrometry (HR-ESI-MS) at *m*/*z* 611.1615 [M − H]^−1^ (the calculated value for C_27_H_31_O_16_ is 611.1612).

**Figure 2:**
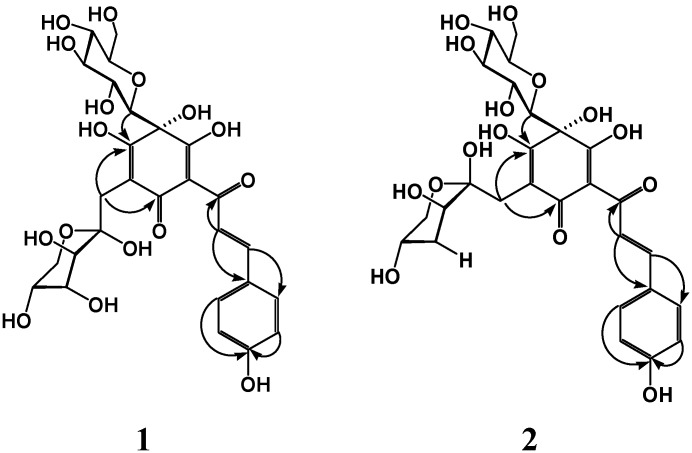
Selected heteronuclear multiple bond correlation (HMBC) for compounds **1** and **2**.

The ^1^H-NMR spectrum of **1** (Table 1) showed *trans* olefinic hydrogen signals at δ 7.45 (1H, d, *J* = 15.5 Hz) and 7.28 (1H, d, *J* = 15.5 Hz), AA'BB' system proton signals at δ 7.43 (2H, d, *J* = 8.5 Hz) and δ 6.82 (2H, d, *J* = 8.5 Hz), and a phenolic hydroxyl proton signal at δ 9.81 (1H, br s), which suggested the existence of a *trans*-*p*-hydroxycinnamoyl group in **1**. Furthermore, two methylene proton signals at δ 2.80 (1H, d, *J* = 14.5 Hz) and 2.64 (1H, d, *J* = 14.5 Hz), an anomeric proton signal at δ 3.79 (1H, d, *J* = 9.0 Hz), two hydroxy proton signal at δ 18.23 (1H, s) and 4.83 (1H, s), and some protons of saccharide moieties at δ 3.0–4.0 ppm were observed. The ^13^C-NMR spectrum of **1** showed 27 carbon signals (Table 1) including a methylene carbon signal at δ 30.2 (C-1'''), three sp^2^ enolic hydroxyl or carbonyl carbons at δ 197.0 (C-3), 190.9 (C-1), and 185.6 (C-5), two sp^2^ quaternary carbons at δ 106.4 (C-2) and 99.7 (C-6), and sp^3^ oxygen-bearing carbon at δ 85.6 (C-4), and nine carbon signals of a *trans*-*p*-hydroxycinnamoyl group in combination with the distortionless enhancement by polarization transfer (DEPT) spectrum. All these pieces of evidence revealed that **1** was a quinochalcone glycoside derivative coupling with absorption maxima at 223, 341, 410 nm in the ultraviolet-visible (UV-vis) spectrum. Comparing the NMR data of compound **1** with those of the corresponding signals of HSYA [4], the significant difference was carbon chemical shifts of a set of carbon signals of the saccharide moiety. The set of carbon signals of the sugar moiety resonating at δ 30.2 (C-1'''), 101.0 (C-2'''), 70.7 (C-3'''), 69.3 (C-4'''), 66.3 (C-5'''), and 62.5 (C-6''') in the ^13^C-NMR spectrum and the carbon signal at δ 30.2 (C-1''') being directly attached to the protons at δ 2.80 (1H, d, *J* = 14.5 Hz) and 2.64 (1H, d, *J* = 14.5 Hz) in the heteronuclear singular quantum correlation (HSQC) spectrum suggested the sugar moiety existed as a α-d-fructopyranose form in **1** [5]. Furthermore, in the heteronuclear multiple bond correlation (HMBC) experiment (Figure 2), the correlations of H-1'''α (δ 2.80) and H-1'''β (δ 2.64) with C-5 (δ 185.6) and C-1 (δ 190.9) revealed that the methylene of the fructopyranose moiety should be directly linked to C-6 of the quinocycle unit. In addition, the correlations of H-1'''α and H-1'''β with H-3''' in the rotating-frame overhauser effect spectroscopy (ROESY) spectrum also indicated that the fructopyranose moiety was α-d-form in combination with the fact that fructose in natural products was D-form [6]. The correlations between H-1"/C-5 and OH-4/C-4, C-1" in the HMBC experiment demonstrated that a glucopyranosyl moiety and a OH group were on C-4 of the quinocycle unit. The C-4 stereochemistry of **1** was elucidated from its circular dichroism (CD) spectroscopic data. In the CD spectrum, **1** exhibited an identical negative cotton effect at around 270 nm with carthamin [7]. Thus the absolute stereochemistry at the C-4 position in **1** was determined to be *S*. Finally, compound **1** was elucidated as 2,5-cyclohexadien-1-one, 4-β-d-glucopyranosyl-6-α-d-fructopyranosyl-3,4,5-trihydroxy-2-[(2*E*)-3-(4-hydroxyphenyl)-1-oxo-2-propenyl]-(9CI), named hydroxysafflor yellow B.

Compound **2** was also obtained as a yellow powder. The IR spectrum of **2** also showed the presence of hydroxyl groups (3444 cm^−1^), carbonyl groups (1635 cm^−1^), and aromatic rings (1513 cm^−1^). The absorption maxima of 2 in the UV-vis spectrum was measured at 224, 341, and 410 nm. The molecular formular of C_27_H_32_O_16_ was deduced on the basis of HR-ESI-MS at *m*/*z* 611.1622 [M − H]^−1^ (the calculated value for C_27_H_31_O_16_ is 611.1612).

**Table 1:** ^1^H-NMR (100 MHz) and ^13^C-NMR (400 MHz) spectroscopic data for compounds **1** and **2**.

No.	1		2	
	δ_C_^(a)^	δ_H_^(b)^ (Mult)	δ_C_^(a)^	δ_H_^(a)^ (Mult)
1	190.9	-	191.5	-
2	106.4	-	106.2	-
3	197.0	18.23 br s (OH)	196.6	-
4	85.6	4.83 br s (OH)	85.4	-
5	185.6	-	184.8	-
6	99.7	-	99.9	-
7	179.7	-	180.4	-
8	120.3	7.28 d (15.5)	120.7	7.29 d (15.5)
9	140.5	7.45 d (15.5)	140.2	7.33 d (15.5)
1'	127.9	-	127.9	-
2', 6'	130.4	7.43 d (8.5)	130.3	7.46 d (8.5)
3', 5'	116.0	6.82 (8.5)	115.9	6.83 d (8.5)
4'	157.7	9.82 br s (OH)	157.6	-
1"	84.9	3.79 overlap	84.9	3.82 overlap
2"	68.5	3.32 m	68.2	3.34 m
3"	77.6	3.31 m	77.5	3.32 m
4"	71.1	3.58 m	71.3	3.56 m
5"	79.1	3.20 m	78.7	3.30 m
6"	59.6	3.76 m 3.68 m	59.6	3.77 m 3.68 m
1'''	30.2	2.80 d (14.5)	31.2	2.85 d (14.5)
		2.64 d (14.5)		2.68 d (14.5)
2'''	101.0	-	100.2	-
3'''	70.7	3.71 m	69.5	3.74 m
4'''	69.3	3.52 m	69.3	3.85 m
5'''	66.3	3.75 m	69.1	3.50 m
6'''	62.5	3.63 m 3.50 m	63.2	3.81 m 3.48 m

^(a)^ Measured in D_2_O; ^(b)^ Measured in DMSO-*d*_6_.

The ^1^H- and ^13^C-NMR spectra were very similar to those of HSYA and **1**, suggesting that **2** also was a quinochalcone glycoside derivative. Major differences form HSYA also came from carbon chemical shifts of a set of carbon signals of the saccharide moiety. The set of carbon signals of the sugar moiety resonating at δ 31.2 (C-1'''), 100.2 (C-2'''), 69.5 (C-3'''), 69.3 (C-4'''), 69.1 (C-5'''), and 63.2 (C-6''') in the ^13^C-NMR spectrum and the carbon signal at δ 31.2 (C-1''') being directly attached to the protons at δ 2.85 (1H, d, *J* = 14.5 Hz) and 2.68 (1H, d, *J* = 14.5 Hz) in the HSQC spectrum suggested the sugar moiety existed as a β-d-fructopyranose form in **2**, rather than α-d-fructopyranose form in **1** [5]. In the HMBC experiment (Figure 2), the correlations of H-1'''α (δ 2.85) and H-1'''β (δ 2.68) with C-5 (δ 184.8) and C-1 (δ 191.5) revealed that the methylene of the fructopyranose moiety should be directly linked to C-6 of the quinocycle unit. The C-4 stereochemistry of **2** was also elucidated from its CD spectroscopic data. In the CD spectrum, **2** also exhibited an identical negative cotton effect at around 270 nm with carthamin [7]. Thus, the absolute stereochemistry at the C-4 position in **2** was determined to be *S*. Finally, compound **2** was elucidated as 2,5-cyclohexadien-1-one, 4-β-d-glucopyranosyl-6-β-d-fructopyranosyl-3,4,5-trihydroxy-2-[(2*E*)-3-(4-hydroxyphenyl)-1-oxo-2-propenyl]-(9CI), named hydroxysafflor yellow C.

**Figure 3:**
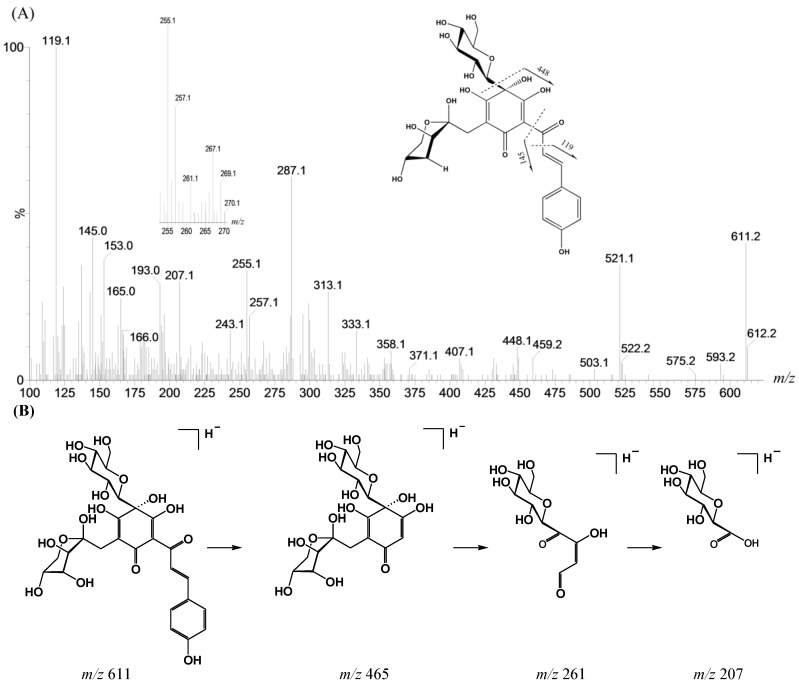
The MS^2^ spectra (**A**), and the fragmentation pathway producing fragment ions at *m*/*z* 261 and 207 in negative ion mode (**B**) (Compound **2** is taken as an example).

High energy collision induced dissociation MS/MS has been shown to be a practical method for structural verification of *C*-glycosyl flavonoids [8]. Studies have shown that the stable carbon–carbon bond of *C*-glycosyl flavonoids is resistant to rupture, so the main cleavages are at the bonds in the sugar moiety, instead of losing a sugar moiety as in *O*-glycosyl flavonoids [9,10]. In the MS/MS spectra of compounds **1** and **2**, both are shown to have lost a glucosyl radical and produce stable radical anions [M − H − 163]^−·^ at *m*/*z* 448. In addition, two fragment ions at *m*/*z* 119 and 145 could be detected in compounds **1** and **2** (Figure 3A), which also proved that they have a *trans*-*p*-hydroxycinnamoyl group. Meanwhile, fragment ions at *m*/*z* 207 and 261 were detected clearly in MS^2^ spectra of compounds **1** and **2** (Figure 3A), which also indicates that they may have a free hydroxyl group at the 4-position [11]. The fragmentation pathway of compound **2** producing fragment ions at *m*/*z* 261 and 207 in negative ion mode is proposed in Figure 3B.

The known compounds were readily identified by comparing their spectroscopic data (UV, IR, ^1^H-NMR, ^13^C-NMR, and HR-ESI-MS) with that reported in the literature as safflomin C (**3**) [12], saffloquinoside C (**4**) [13], and (2*R*)-4',5-dihydroxyl-6,7-di-*O*-β-d-glucopyranosyl flavanone (**5**) [14].

**Figure 4:**
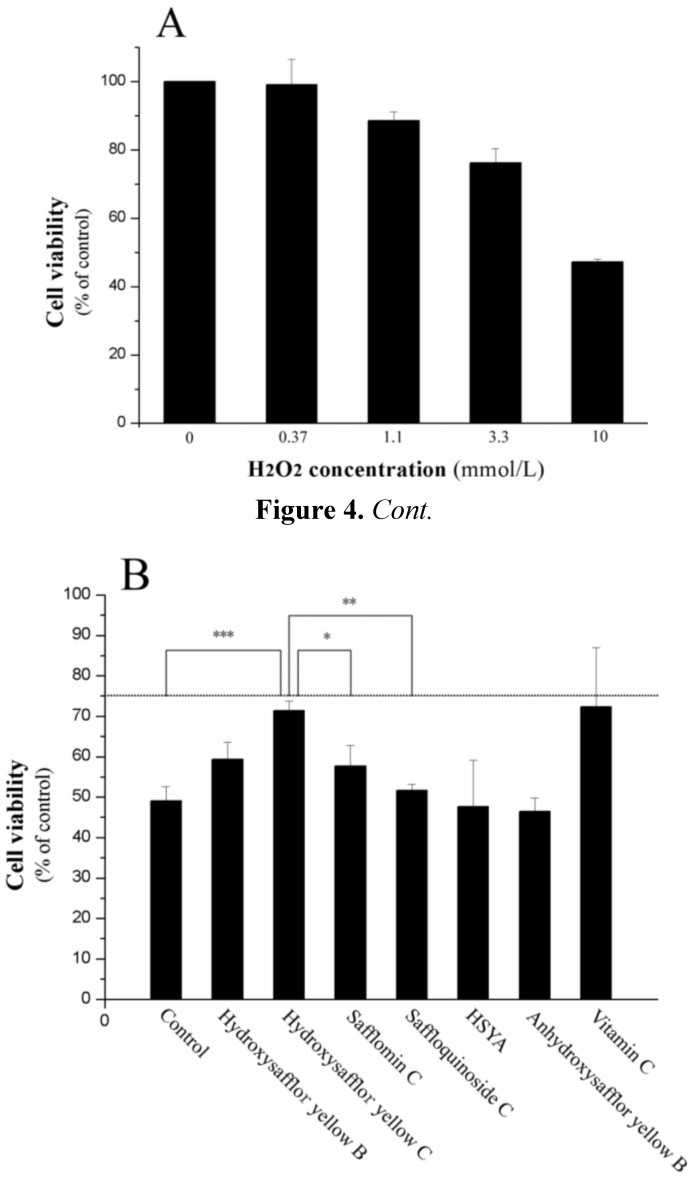
*In vitro* anti-oxidative effects of the isolated quinochalcone *C*-glycosides against H_2_O_2_-induced cytotoxicity in cultured H9c2 cells. (**A**) Various concentrations of H_2_O_2_ (0, 0.37, 1.1, 3.3 and 10 mmol/L) were added onto cultured H9c2 cells, incubated for 1 h and determined with cell viability; (**B**) The isolated quinochalcone *C*-glycosides (60 μg/mL) were pretreated with H9c2 cells for 24 h before the addition of H_2_O_2_ (10 mmol/L) for cytotoxicity test as in (**A**). Vitamin C (1.1 mg/mL) served as a positive control (statistical significance is indicated as * *p* = 0.031 for hydroxysafflor yellow C *vs.* safflomin C; ** *p* = 0.005 for hydroxysafflor yellow C *vs.* saffloquinoside C; *** *p* = 0.001 for hydroxysafflor yellow C *vs.* control).

The obtained quinochalcone *C*-glycosides together with HSYA and anhydrosafflor yellow B were evaluated for their anti-oxidative effects against H_2_O_2_-induced cytotoxicity in cultured H9c2 cells (Figure 4). Among them, the cardioprotective effects of compound **2** were more robust than those of compounds **1**, **3** and **4**. On the other hand, HSYA and anhydrosafflor yellow B did not show any significant response.

### 2.2. Discussion

Quinochalcone *C*-glycosides are the typical constituents of *Carthamus tinctorius*. Two new quinochalcone *C*-glycosides (**1**–**2**), which add the diversity of these ingredients, were successfully isolated from safflower. The NMR and MS/MS analyses led to elucidation of the structure of compounds **1** and **2** with their partial configurations. Moreover, for d-fructose, the equilibrium concentrations in water are around 57% of β-d-pyranose, 31% of β-d-furanose, 9% of α-d-furanose, and 3% of α-d-pyranose [15]. Coincidentally, we isolated much less of compound **1** than compound **2** from safflower. In the total synthesis of quinochalcone *C*-glycosides, asymmetric synthesis of carthamin (as the acetate) has been achieved [7]. However, the total syntheses of the other yellow pigments of quinochalcone *C*-glycosides have not been carried out. The synthesis of analogs in which the glucosyl group or the glucosyl and hydroxyl groups on the chiral carbon were replaced by one or two methyl groups has been achieved for safflomin A, safflomin C, precarthamin, and carthamin [2]. So the study may provide some inspiration for scientists to explore the biogenesis and the total synthesis of quinochalcone *C*-glycosides.

H_2_O_2_ has been extensively used as an inducer of oxidative stress *in vitro* [16]. Meanwhile, the H9c2 cell line has been widely used in studies investigating cardiomyocyte cellular mechanisms [17,18]. It is worth mentioning that the water extract of *Carthamus tinctorius* has been developed as an intravenous injection in China and has been extensively applied in hospitals to treat cardiovascular diseases [19]. Thus, it is of interest to determine whether the isolated quinochalcone *C*-glycosides against H_2_O_2_-induced cytotoxicity in cultured H9c2 cells. Of the compounds isolated, the cardioprotective effects of compound **2** were more robust than those of compounds **1**, **3** and **4**, thereby providing a basis for further studies. On the other hand, HSYA and anhydrosafflor yellow B did not show any significant response.

## 3. Experimental Section

### 3.1. General Experimental Procedures

The optical rotations were measured on a Jasco P-1020 polarimeter (Jasco, Tokyo, Japan). IR spectra were recorded on a Bruker Tensor 27 FT-IR (Bruker, Ettlingen, Germany). The ^1^H- and ^13^C-NMR and 2D NMR spectra were recorded on Bruker Avance 400 spectrometers using tetramethylsilane (TMS) as an internal standard. The HR-ESI-MS data were obtained using a Synapt MS Q-TOF (Waters Corp, Milford, MA, USA). Column chromatography (CC) was performed on macroporous adsorbent resin D101 (0.3–1.25 mm; Qingdao Marine Chemical Company, Qingdao, China), Sephadex LH-20 (40–70 μm; GE Healthcare Bio-Sciences AB, Uppsala, Sweden). Reversed-phase preparative high-performance liquid chromatography (HPLC) was performed on a Waters Auto Purification System equipped with a UV-vis detector and a XBridge™ C18 OBD™ column (150 × 30 mm, 5 μm).

### 3.2. Plant Material

The florets of *Carthamus tinctorius* were purchased from Tongling TCM Co., Ltd., Fengyuan, Anhui Province in March 2013 and identified by Professor Chun-Gen Wang. A voucher specimen (No. NJUTCM-20130308) was deposited in the Herbarium of Nanjing University of Chinese Medicine.

### 3.3. Extraction and Isolation

The dried florets of *Carthamus tinctorius* (15 kg) were percolated with 95% EtOH and 70% EtOH (*v*/*v*) to colorless, respectively. Then the combined EtOH extracts were concentrated under reduced pressure to yield a brownish extract (1950 g) that was suspended in H_2_O. The suspension was partitioned successively with petroleum ether (60–90 °C) and EtOAc (eight times each). The surplus suspension was then chromatographed over macroporous adsorbent residue (D101) column. After eluting with H_2_O, the adsorbed constituents were eluted with 5% EtOH, 30% EtOH, 50% EtOH, and 95% EtOH, respectively. The 30% EtOH part was chromatographed over Sephadex LH-20, eluting with H_2_O-MeOH (from 100:0 to 0:100) to give 30 fractions. Fr.3, Fr.5, Fr.6 and Fr.11 were further purified by reversed-phase preparative HPLC with to yield **3** (120 mg), **2** (350 mg), **1** (40 mg), **5** (20 mg), and **4** (10 mg), respectively. The mobile phase was composed of solvent A (0.1% aqueous formic acid, *v*/*v*) and B (methanol), the gradient program was set as follows: 16%–19% B at 0–5 min, 19% B at 5–15 min, 19%–44% B at 15–20 min, 44% B at 20–30 min, 44%–100% B at 30–32 min. The flow rate was 15 mL/min and the detection wavelength was 407 nm.

#### 3.3.1. Hydroxysafflor Yellow B (**1**)

Yellow powder. [α]_D_^26^: −181.4 (*c* 0.1, MeOH). UV (MeOH): λ_max_ = 223, 341, 410 nm. IR (KBr): υ_max_ = 3368 (OH), 1651 (C=O) cm^−1^. For ^1^H- and ^13^C-NMR spectroscopic data, see Table 1. HR-ESI-MS: *m*/*z* = 611.1615 [M − H]^−1^ (calcd for C_27_H_31_O_16_, 611.1612).

#### 3.3.2. Hydroxysafflor Yellow C (**2**)

Yellow powder. [α]_D_^26^: −175.0 (*c* 0.15, MeOH). UV (MeOH): λ_max_ = 224, 341, 410 nm. IR (KBr): υ_max_ = 3368 (OH), 1651 (C=O) cm^−1^. For ^1^H- and ^13^C-NMR spectroscopic data, see Table 1. HR-ESI-MS: *m*/*z* = 611.1622 [M − H]^−1^ (calcd for C_27_H_31_O_16_, 611.1612).

#### 3.3.3. Supplementary Files

MS, HRMS, IR, 1D and 2D NMR of compounds **1** and **2** are available as Supplementary Information.

### 3.4. Biological Assay

The anti-oxidative effects of the isolated quinochalcone *C*-glycosides were evaluated against H_2_O_2_-induced cytotoxicity in cultured H9c2 cells. The purity of the tested compounds was >98% as identified by NMR and MS.

#### 3.4.1. Cell Culture

Rat pheochromatocytoma H9c2 cell line was obtained from ATCC (Rockefeller, MD, USA). The cells were maintained in Dulbecco’s modified Eagles medium (DMEM) supplemented with 10% fetal bovine serum at 37 °C in a water-saturated 5.0% CO_2_ incubator. Reagents for cell cultures were purchased from Sigma (St. Louis, MO, USA).

#### 3.4.2. Cell Viability Test

Cultured H9c2 cells in 96-well-plate (6000 cells/well) were pre-treated with the quinochalcone *C*-glycosides (60 μg/mL) for 24 h. After being replaced by fresh culture medium, the cultures were treated with 10 mmol/L hydrogen peroxide (H_2_O_2_) for 1 h. Cell viability tests were performed with the addition of thiazolyl blue tetrazolium bromide (MTT) (Sigma, St. Louis, MO, USA) in fresh water at a final concentration of 5 mg/mL for three hours. After the solution was removed, the purple preciptate inside the cells was re-suspended in DMSO and then measured at 570 nm absorbance. H_2_O_2_ at various concentrations (0, 0.37, 1.1, 3.3 and 10 mmol/L) served as a control for cytotoxicity.

#### 3.4.3. Statistical Analysis

Individual data were expressed as mean ± standard deviation (SD). A *post-hoc* Dunnett’s test was used to obtain corrected *p*-values in group comparisons. Statistical analyses were performed with one-way ANOVA (SPSS version 17.0: Chicago, IL, USA). A *p* value of 0.05 or less was considered significant.

#### 3.4.4. Sample Availability

Samples of the compounds **1** and **2** are available from the authors.

## 4. Conclusions

In recent years, *C*-glycoside chemistry has been one of the main topics in carbohydrate chemistry, not only because of the synthetic challenges posed, but also because *C*-glycosides have the potential to serve as carbohydrate analogues resistant to metabolic processes. Consequently, *C*-glycosides are currently receiving much interest as a potential source of therapeutic agents for clinical use [20]. Quinochalcone *C*-glycosides are regarded as the characteristic compounds that only have been isolated from the florets of *Carthamus tinctorius*. Recently, this class of compounds was found to have multiple pharmacological activities. Except for the water extract of *Carthamus tinctorius*, it is worth mentioning that HSYA also has been developed as an intravenous injection in China to treat cardiovascular diseases. In the study, two new quinochalcone *C*-glycosides, hydroxysafflor yellow B (**1**) and hydroxysafflor yellow C (**2**) were isolated and identified from the florets of *Carthamus tinctorius*. To the best of our knowledge, the *C*-α-d-fructopyranose is rare in natural quinochalcone *C*-glycosides. Previous studies have found that quinochalcone *C*-glycosides are soluble in water, diluted alcohol and practically insoluble in anhydrous ethanol, acetone, diethyl ether, petroleum, and ethyl acetate [21]. However, from our experience, there are large differences in the polarity for this class of compounds, which results in difficulty in NMR experiments. Once the molecular weight of quinochalcone *C*-glycosides exceeds 1000, simple signal accumulation does not work for ^13^C-NMR measurement, which prompted us to develop a better approach and strategy. Anti-oxidative activity evaluation showed that compound **2** was significantly active against H_2_O_2_-induced cytotoxicity in cultured H9c2 cells *in vitro*, which suggests its potential for treatment of cardiovascular diseases.

## Supplementary Materials

Supplementary Figures can be found at http://www.mdpi.com/1422-0067/15/9/16760/s1.

## Supplementary Files

Supplementary File 1
