# Supplementary material for: Two New Quinochalcone C-Glycosides from the Florets of Carthamus tinctorius
Source: Int J Mol Sci. 2014 Sep 22;15(9):16760–71. doi: 10.3390/ijms150916760 (PMC4200872; doi:10.3390/ijms150916760)

Supplementary Information

Figure S1. IR spectrum of hydroxysafflor yellow B (**1**).

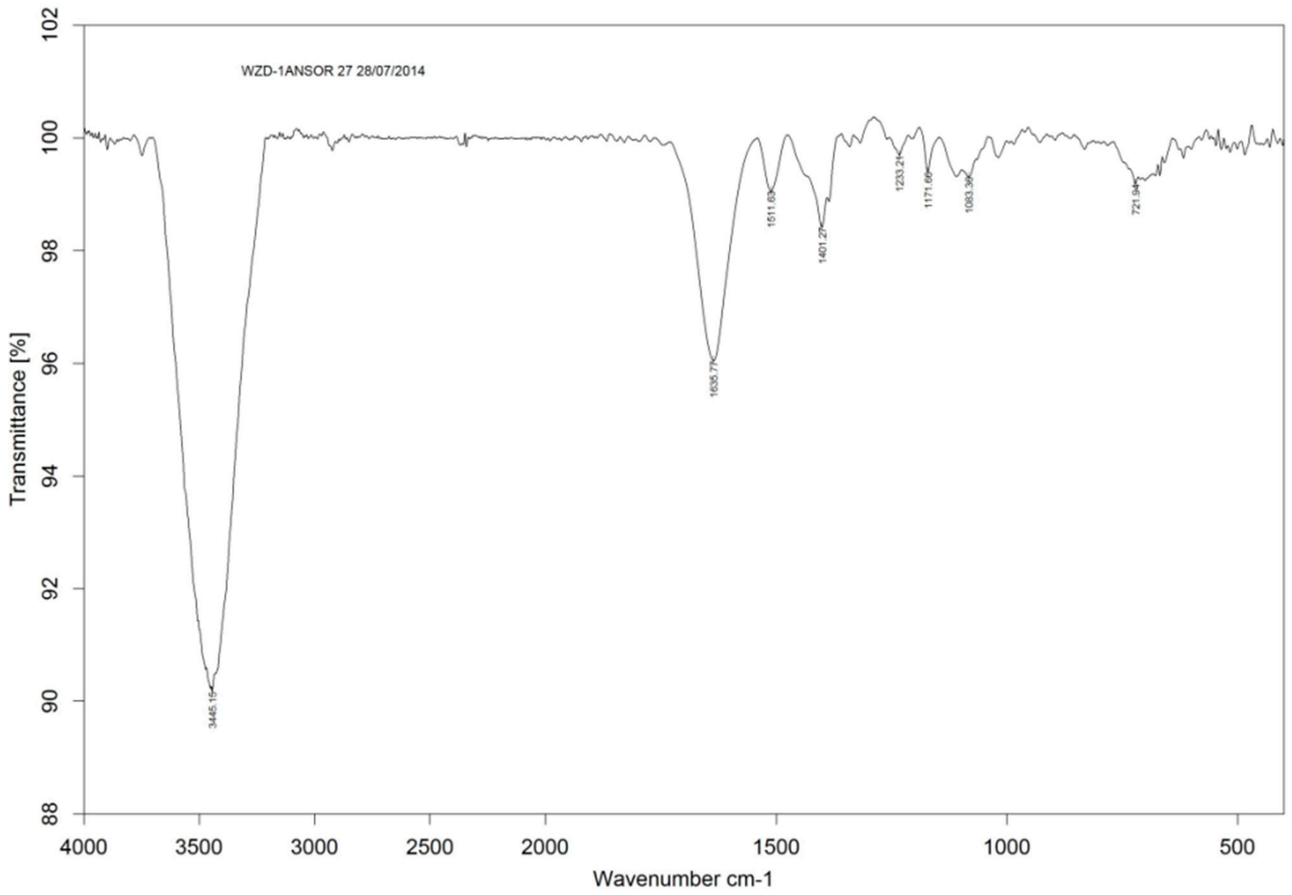

Figure S2. ESI spectrum of hydroxysafflor yellow B (**1**) in MeOH.

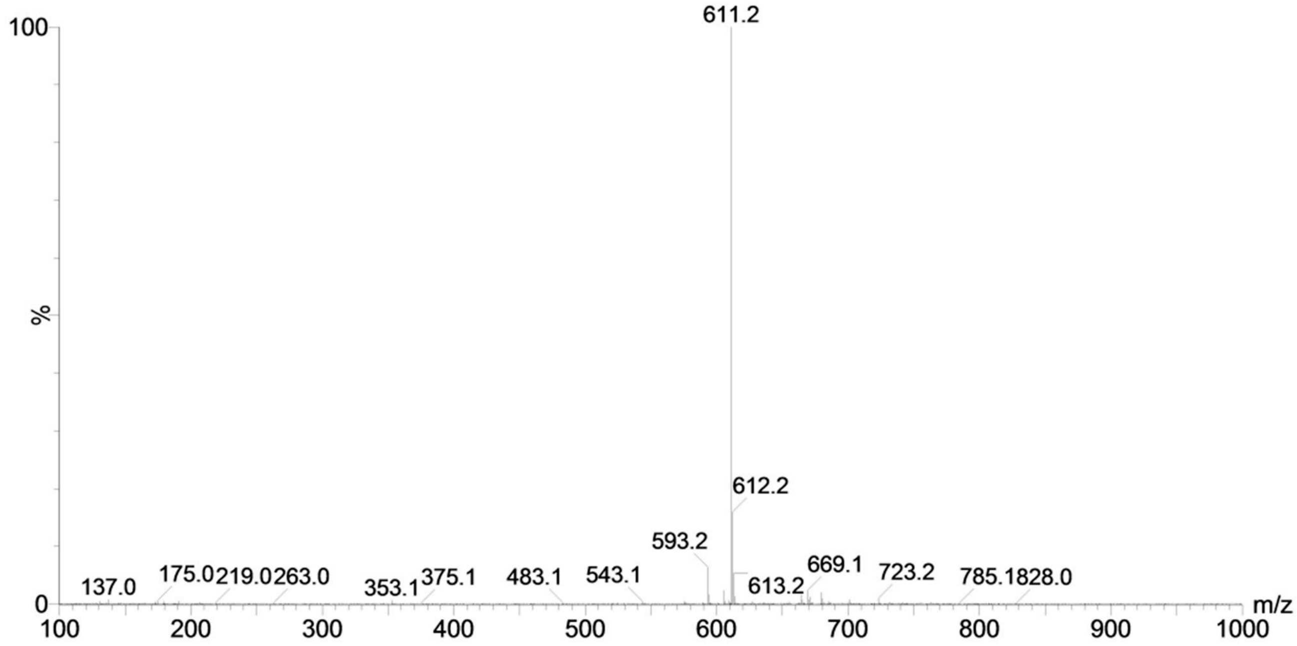

Figure S3. HRESIMS spectrum of hydroxysafflor yellow B (**1**) in MeOH.

Monoisotopic Mass, Odd and Even Electron Ions
186 formula(e) evaluated with 2 results within limits (up to 50 best isotopic matches for each mass)
Elements Used:
C: 0-500 H: 0-1000 O: 0-200
1
leshijun_140812_5 71 (2.176) 2: TOF MSMS 611.00ES- 1.80e+002

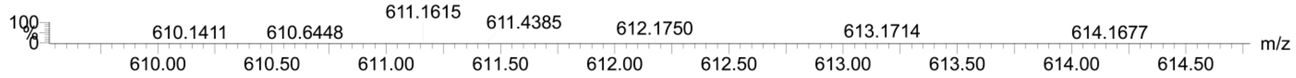

Minimum:	80.00				-1.5				
Maximum:	100.00		5.0	10.0	50.0				
Mass	RA	Calc. Mass	mDa	PPM	DBE	i-FIT	Norm	Conf(%)	Formula
611.1615	100.00	611.1612	0.3	0.5	12.5	12.9	0.044	95.71	C27 H31 O16
		611.1647	-3.2	-5.2	34.5	16.0	3.150	4.29	C45 H23 O3

Figure S4. ^{1}H-NMR spectrum of hydroxysafflor yellow B (**1**) in DMSO-d_6.

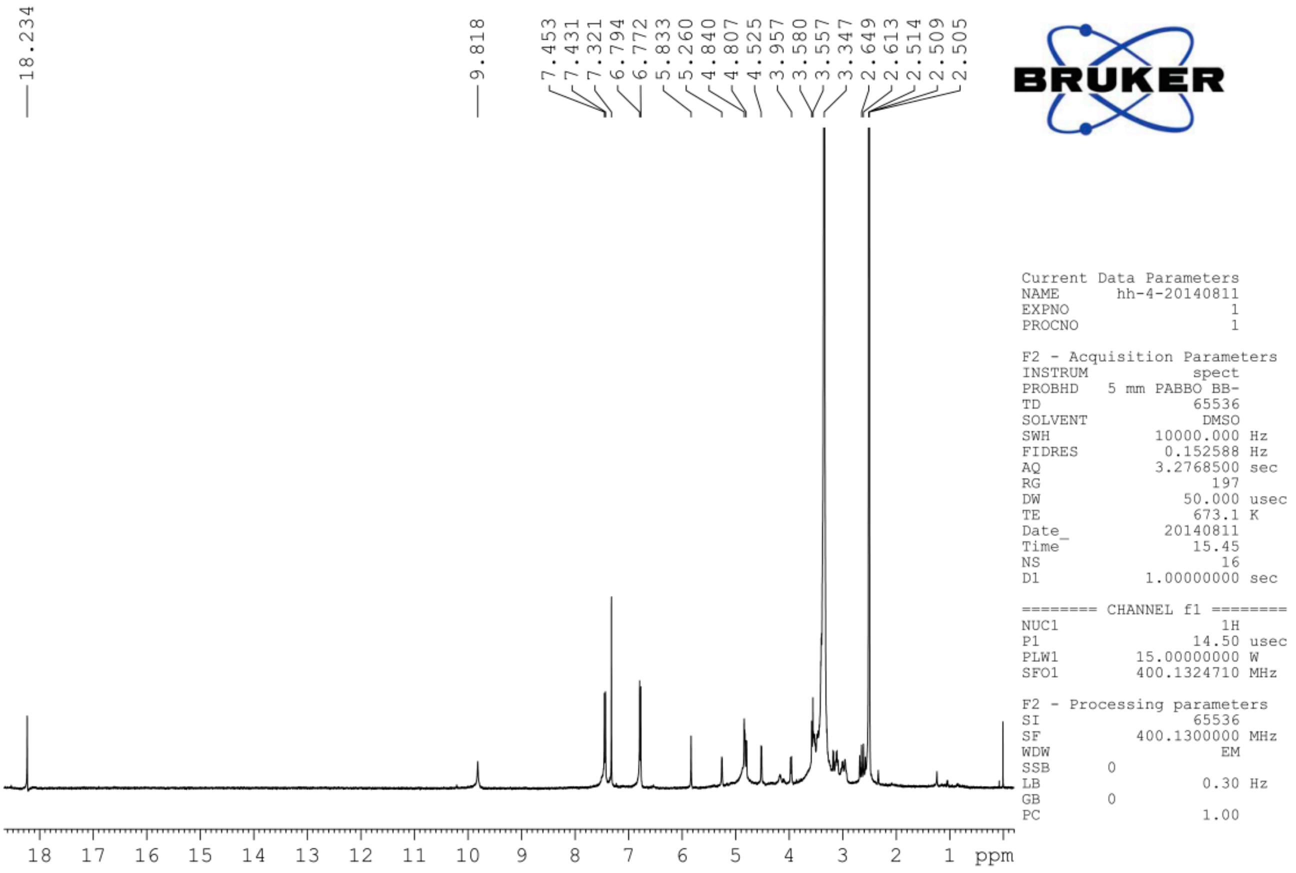

Figure S5. ^{1}H-NMR spectrum of hydroxysafflor yellow B (**1**) in D_2O.

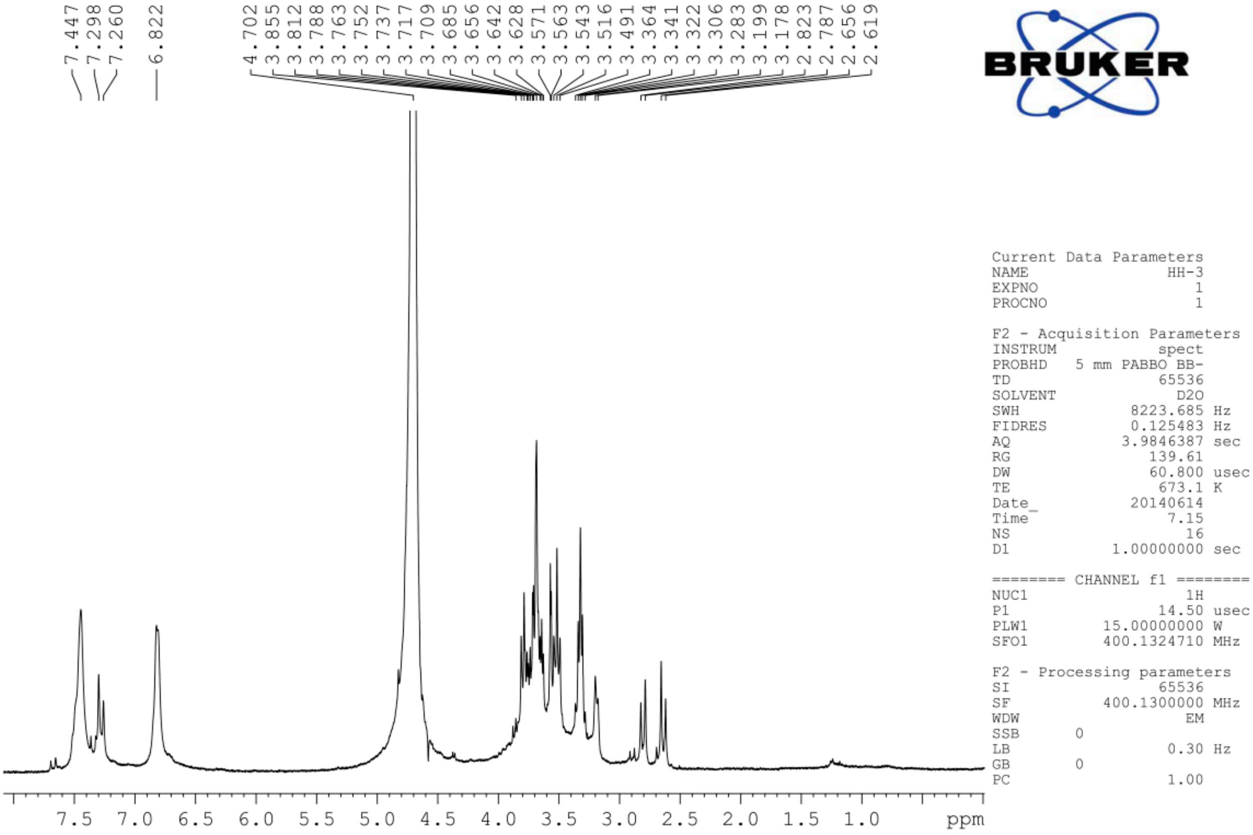

Figure S6. ^{13}C-NMR spectrum of hydroxysafflor yellow B (**1**) in D_2O.

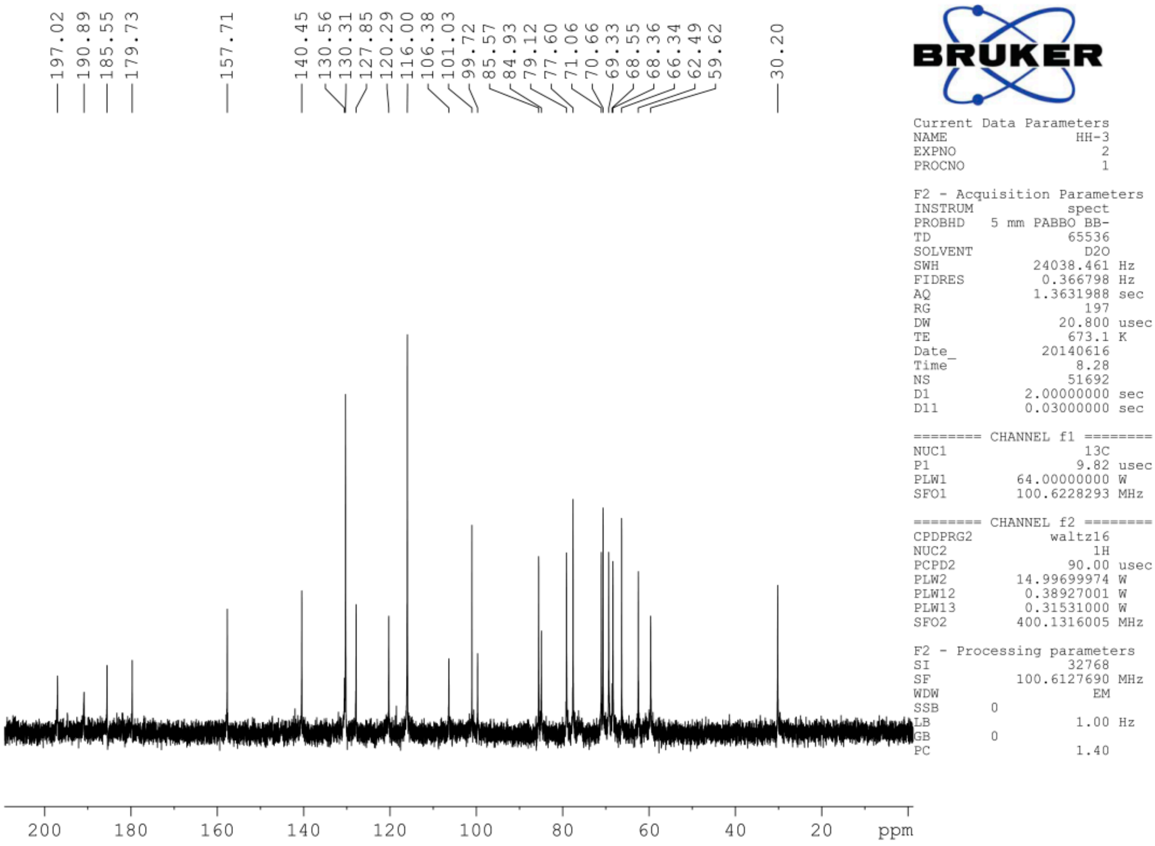

Figure S7. HSQC NMR spectrum of hydroxysafflor yellow B (**1**) in D_2O.

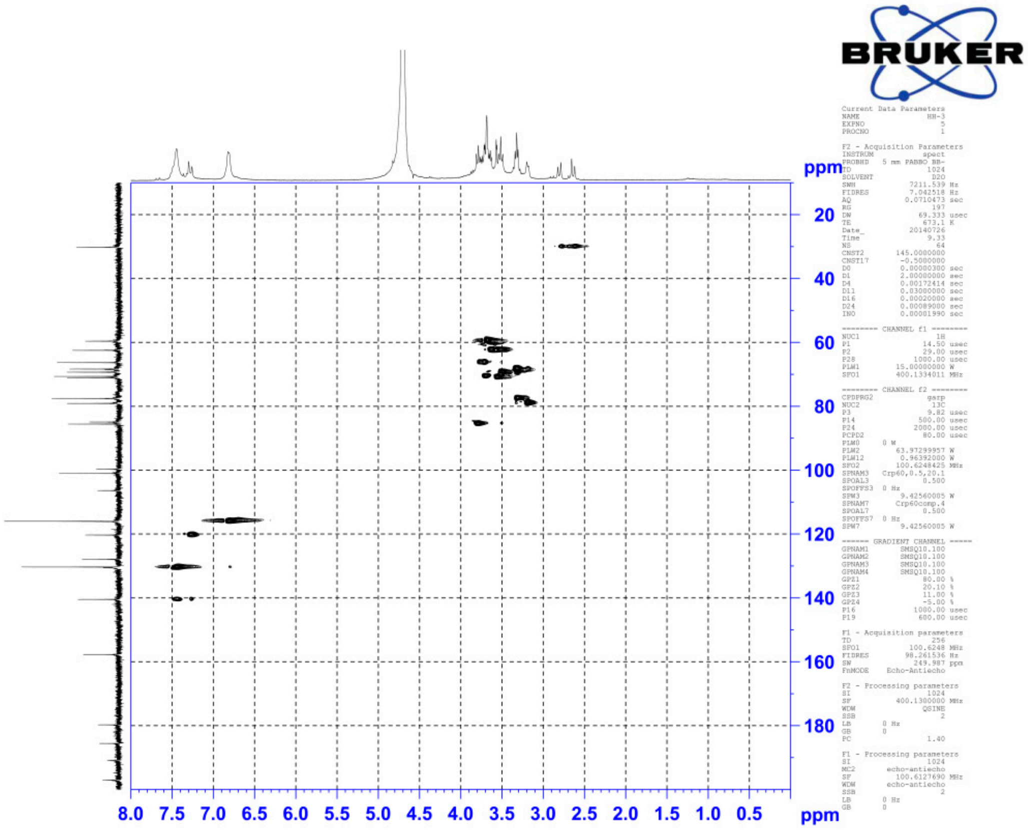

Figure S8. HMBC NMR spectrum of hydroxysafflor yellow B (**1**) in D_2O.

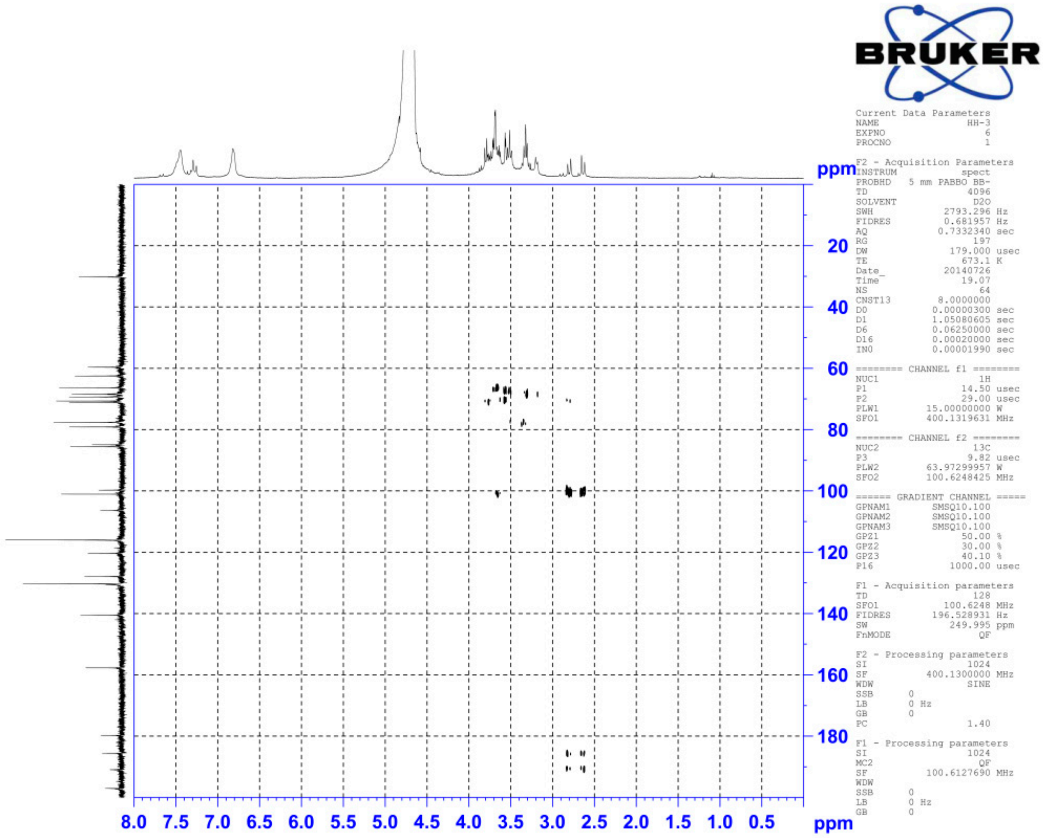

Figure S9. COSY NMR spectrum of hydroxysafflor yellow B (**1**) in D_2O.

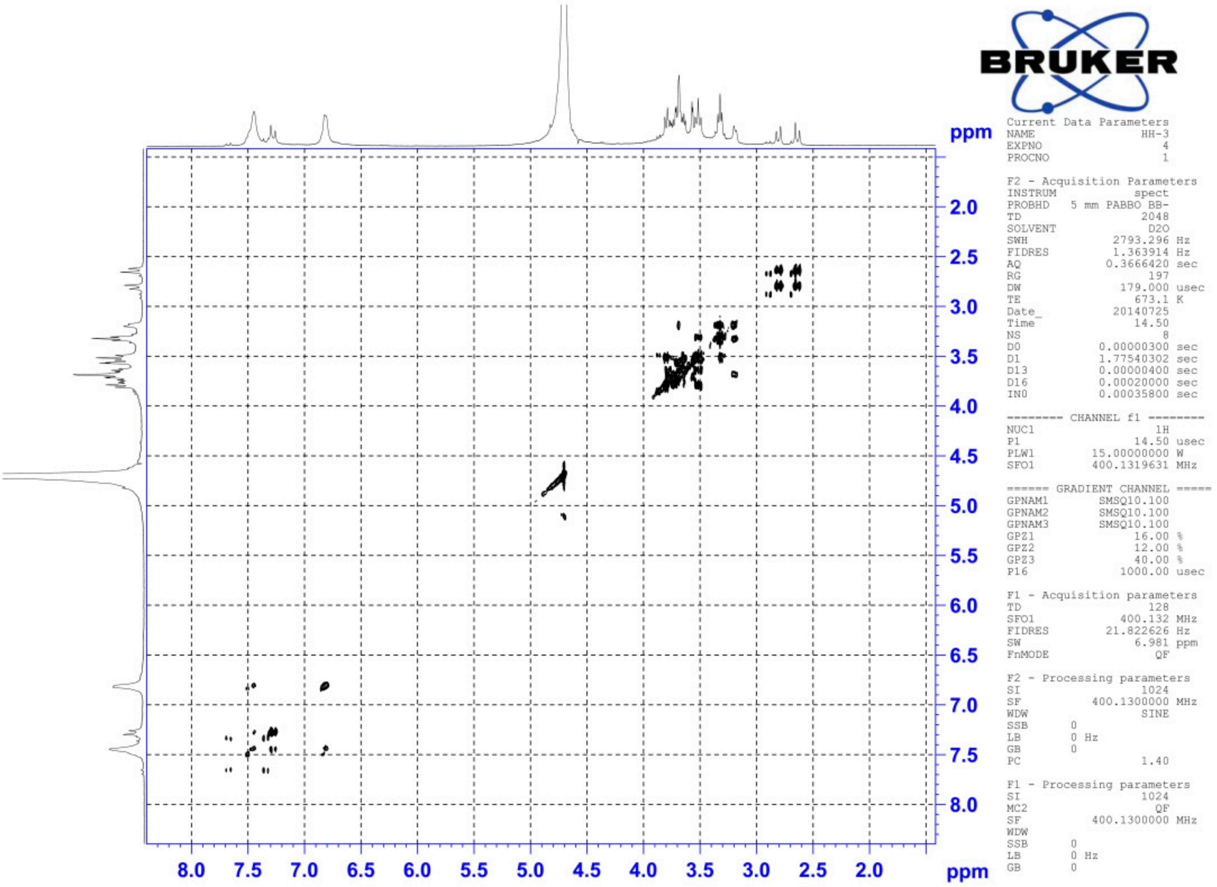

Figure S10. IR spectrum of hydroxysafflor yellow C (**2**)

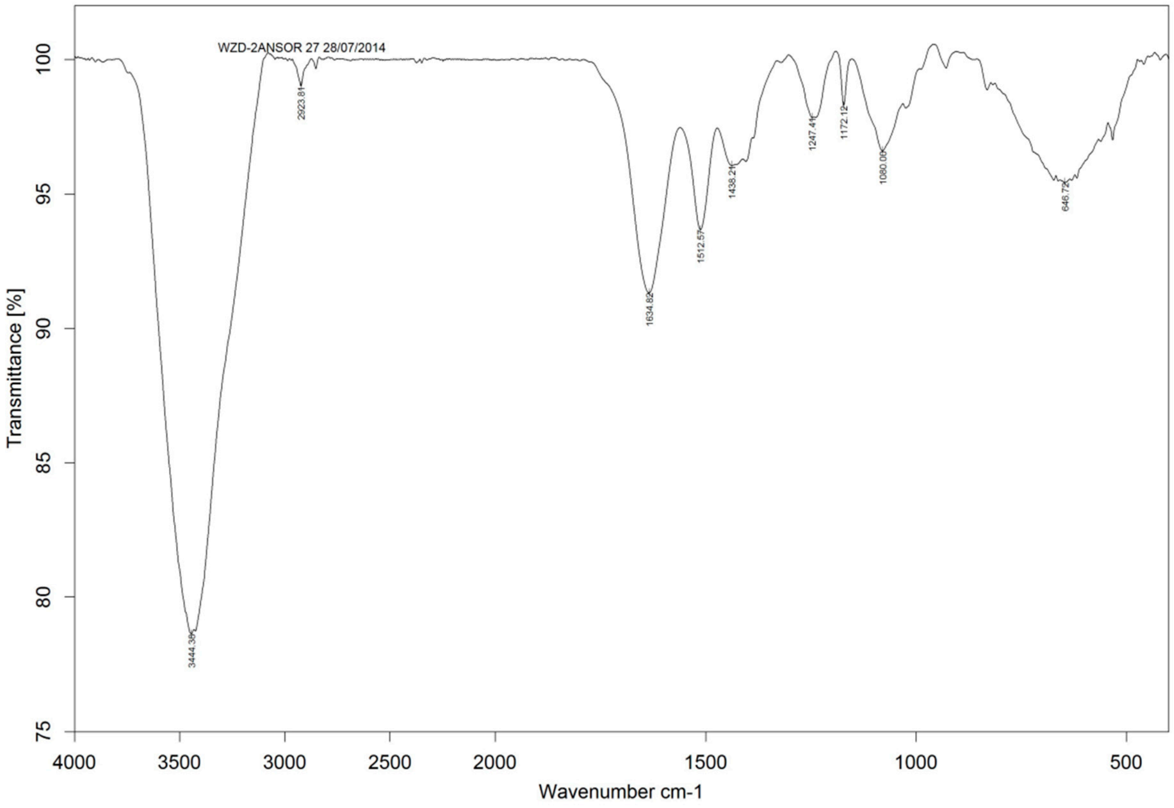

Figure S11. ESI spectrum of hydroxysafflor yellow C (**2**) in MeOH.

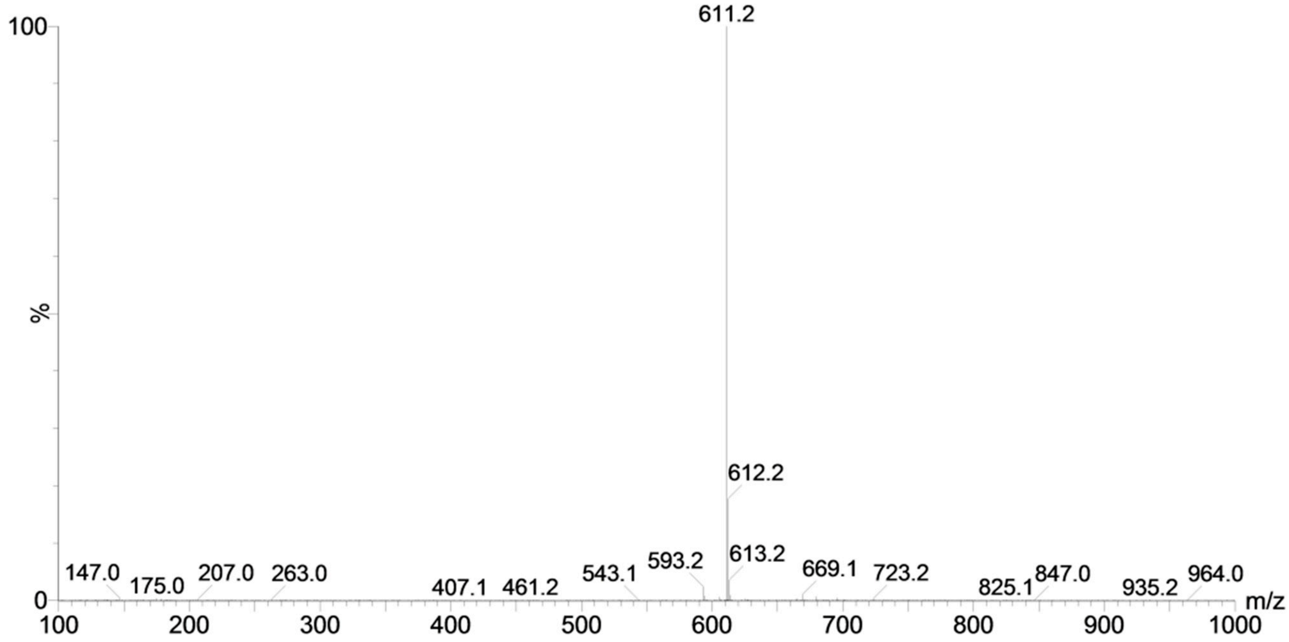

Figure S12. HRESIMS spectrum of hydroxysafflor yellow C (**2**) in MeOH.

Monoisotopic Mass, Odd and Even Electron Ions
186 formula(e) evaluated with 3 results within limits (up to 50 best isotopic matches for each mass)
Elements Used:
C: 0-500 H: 0-1000 O: 0-200
2
leshijun_140812_6 70 (2.148)

2: TOF MSMS 611.00ES-
8.90e+001

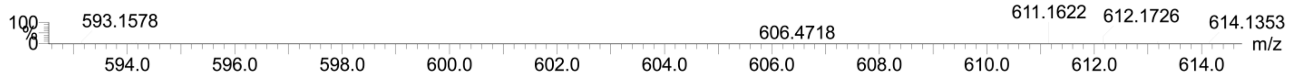

Minimum:	80.00				-1.5				
Maximum:	100.00		5.0	10.0	50.0				
Mass	RA	Calc. Mass	mDa	PPM	DBE	i-FIT	Norm	Conf(%)	Formula
611.1622	100.00	611.1612	1.0	1.6	12.5	10.0	0.716	48.88	C27 H31 O16
		611.1671	-4.9	-8.0	3.5	10.3	1.087	33.72	C20 H35 O21
		611.1647	-2.5	-4.1	34.5	11.0	1.748	17.41	C45 H23 O3

Figure S13. ^{1}H-NMR spectrum of hydroxysafflor yellow C (**2**) in D_2O.

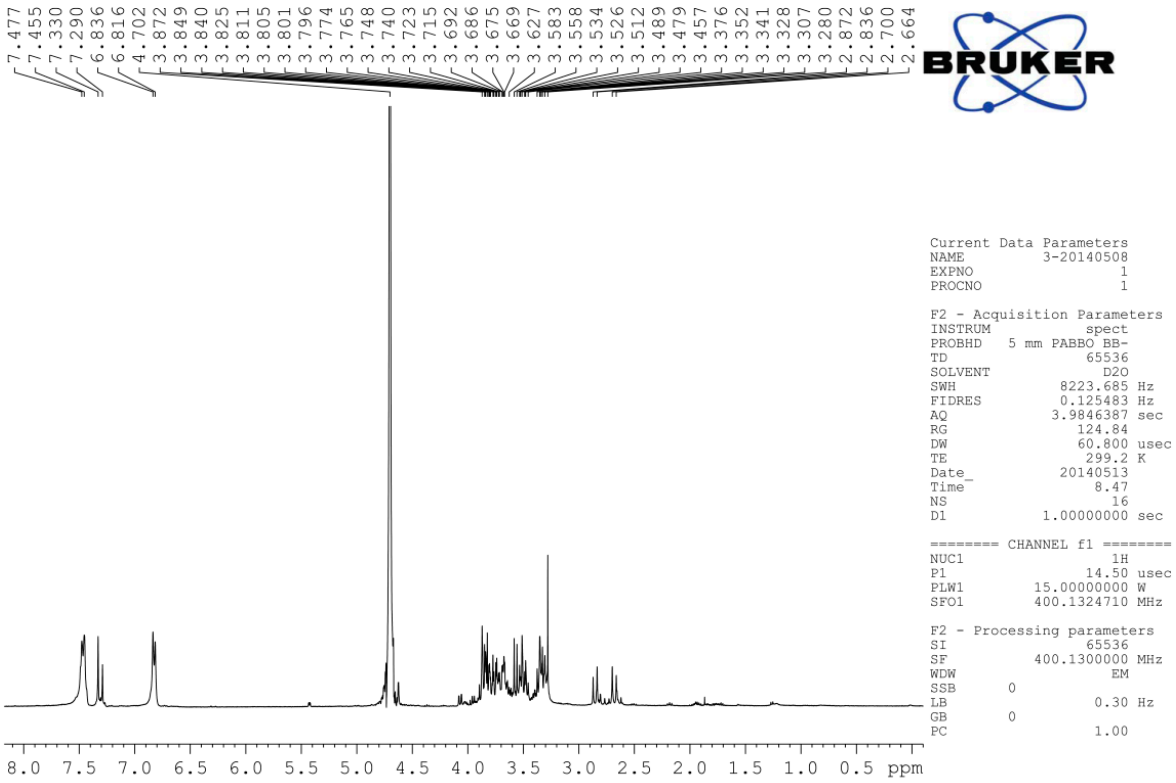

Figure S14. ^{13}C-NMR spectrum of hydroxysafflor yellow C (**2**) in D_2O.

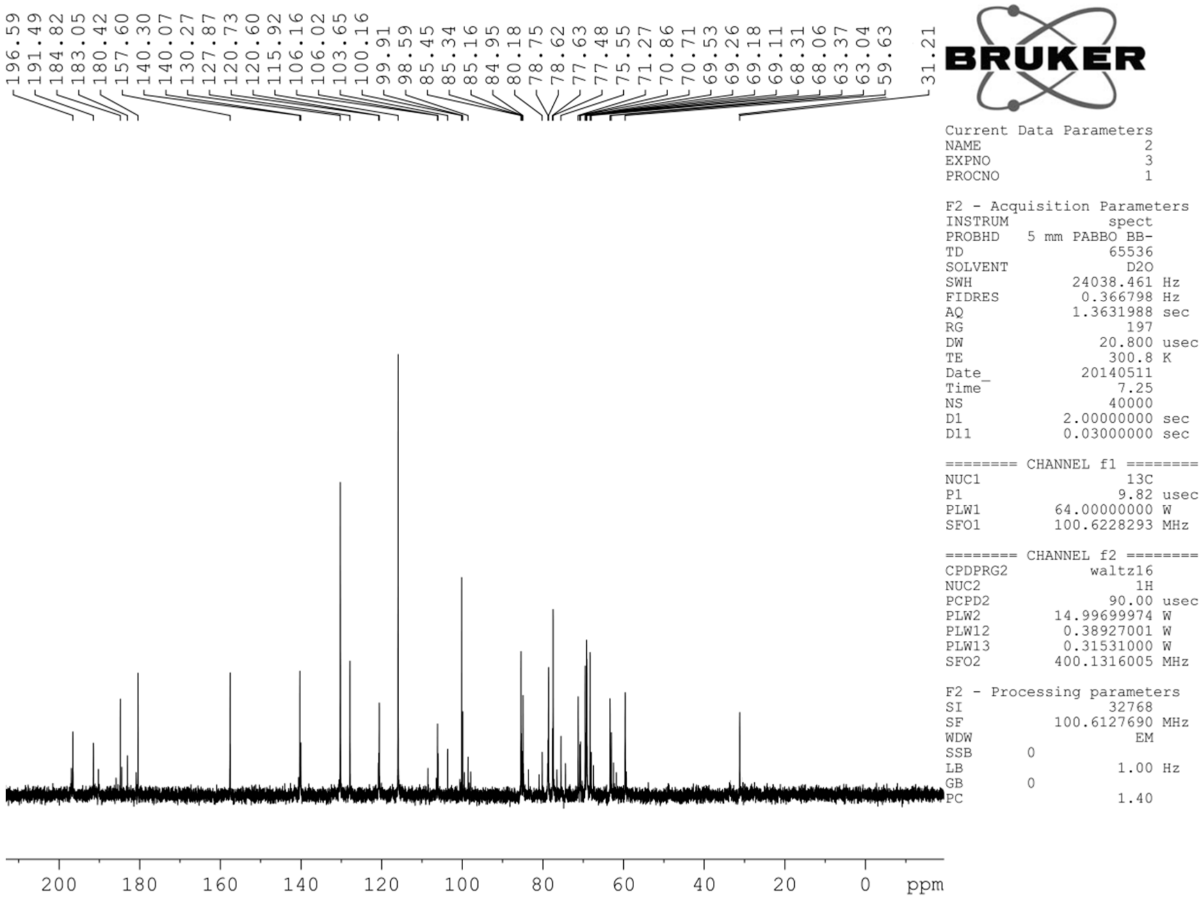

Figure S15. HSQC NMR spectrum of hydroxysafflor yellow C (**2**) in D_2O.

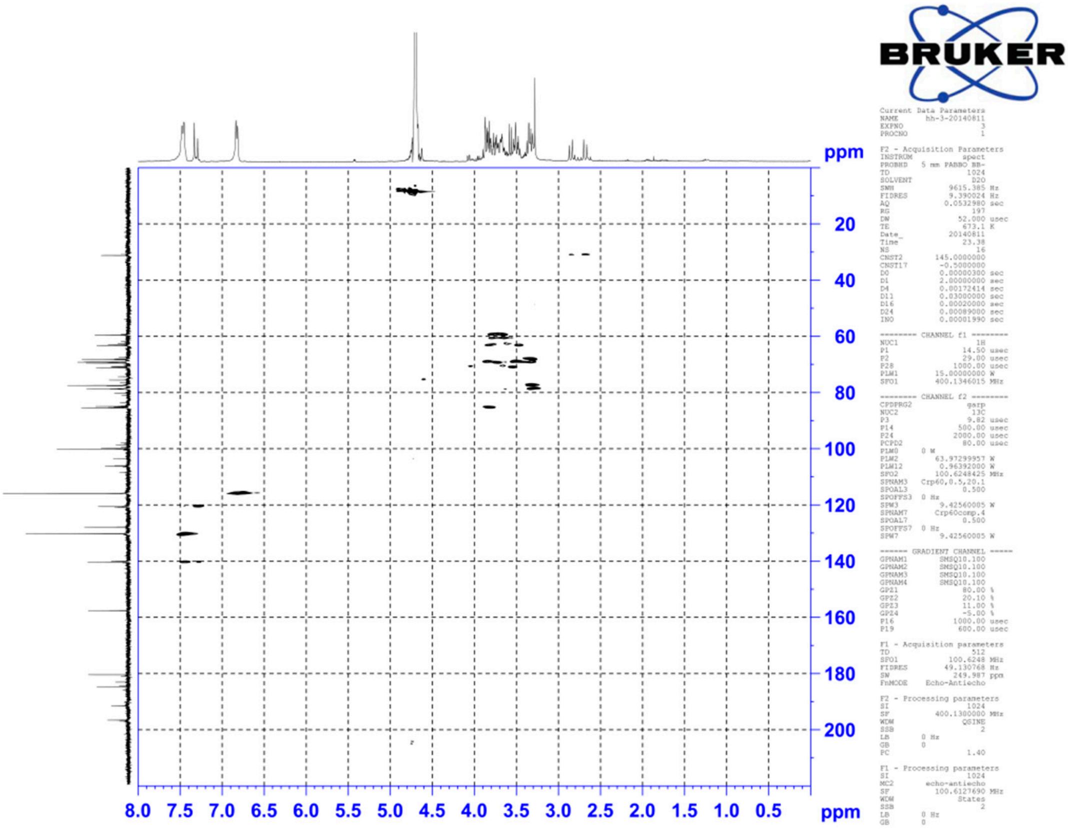

Figure S16. COSY NMR spectrum of hydroxysafflor yellow C (**2**) in D_2O.

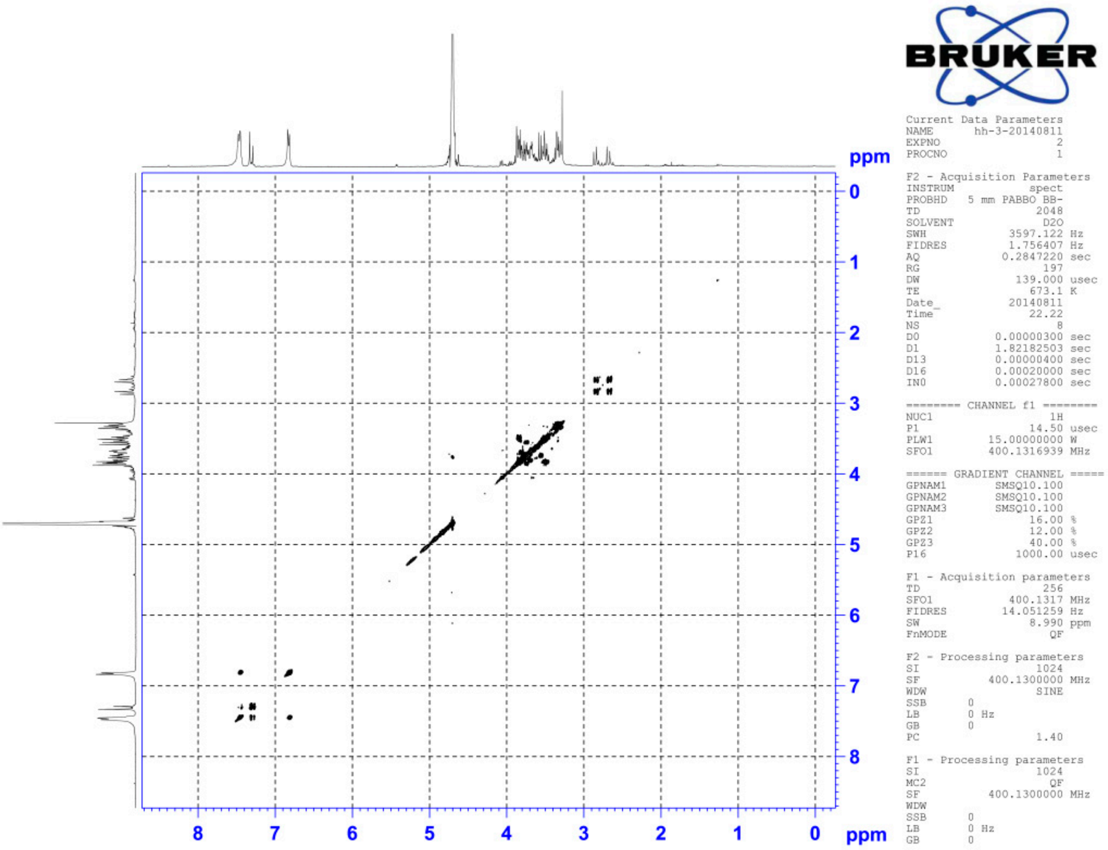

Figure S17. ^{1}H NMR spectrum of saffloquinoside C (**4**) in D_2O.

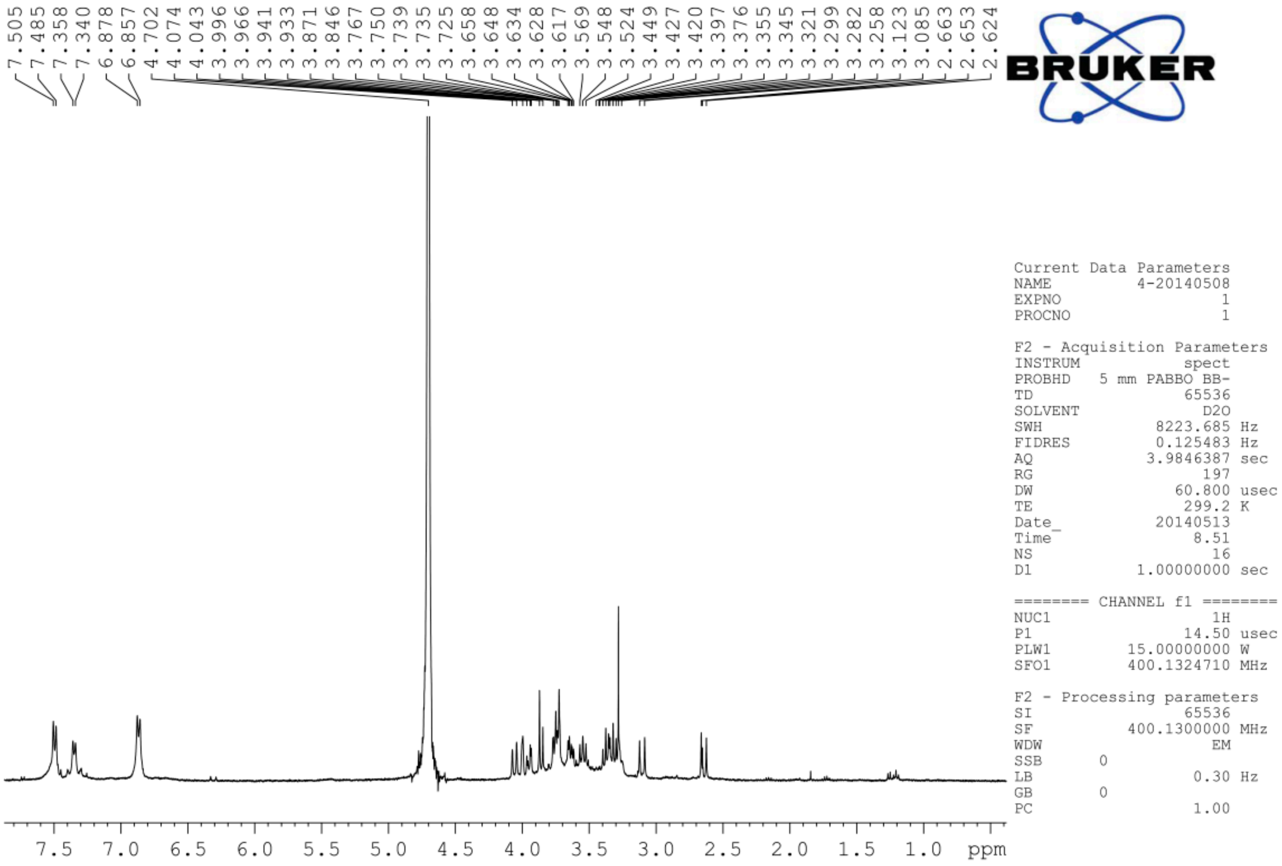

Supplement: Supplementary File 1 [file ijms-15-16760-s001.pdf]
